# A Two-Piece Derivative of a Group I Intron RNA as a Platform for Designing Self-Assembling RNA Templates to Promote Peptide Ligation

**DOI:** 10.1155/2012/305867

**Published:** 2012-08-22

**Authors:** Takahiro Tanaka, Hiroyuki Furuta, Yoshiya Ikawa

**Affiliations:** ^1^Department of Chemistry and Biochemistry, Graduate School of Engineering, Kyushu University, Motooka 744, Nishi-ku, Fukuoka 819-0395, Japan; ^2^International Research Center for Molecular Systems, Kyushu University, Motooka 744, Nishi-ku, Fukuoka 819-0395, Japan; ^3^Synthetic Systems Biology Research Center, Kyushu University, Maidashi 3-1-1, Higashi-ku, Fukuoka 812-8582, Japan

## Abstract

Multicomponent RNA-peptide complexes are attractive from the viewpoint of artificial design of functional biomacromolecular systems. We have developed self-folding and self-assembling RNAs that serve as templates to assist chemical ligation between two reactive peptides with RNA-binding capabilities. The design principle of previous templates, however, can be applied only to limited classes of RNA-binding peptides. In this study, we employed a two-piece derivative of a group I intron RNA from the *Tetrahymena* large subunit ribosomal RNA (LSU rRNA) as a platform for new template RNAs. In this group I intron-based self-assembling platform, modules for the recognition of substrate peptides can be installed independently from modules holding the platform structure. The new self-assembling platform allows us to expand the repertoire of substrate peptides in template RNA design.

## 1. Introduction

In modern cellular systems, RNAs frequently associate with proteins to form ribonucleoprotein (RNP) complexes [1–5]. In these RNP complexes, biological functions ascribed to the main component (RNA or protein) are regulated by the accessory component (protein or RNA). During molecular evolution, both main and accessory components often evolved from single- to multi-molecular systems to elaborate and diversify their functions. A typical example can be seen in ribonuclease P (RNaseP), which plays a pivotal role in tRNA processing [6–8]. Most RNaseP enzymes act as ribonucleoproteins (RNPs), which have been proposed to have evolved from a catalytic RNA. While prokaryotic RNaseP is composed of a single RNA subunit and single protein subunit [6], eukaryotic RNaseP has 9-10 protein subunits that decorate one RNA subunit [6–8]. A different type of multicomponent system is seen in the evolutionary fragmentation of RNA components in *Caulobacter* and *Euglena*, in which the tmRNA [9] and large ribosomal RNA [10] are reconstituted by assembling 2 and 14 fragments, respectively.

Multicomponent systems have advantages in functional diversification of biomolecular complexes. For example, it is well known that some protein subunits of eukaryotic RNase P are shared with RNase MRP, which processes non-tRNA substrates [11, 12]. More recently, the core proteins of telomerase RNP complex were reported to play novel roles by associating with nontelomerase RNA [13, 14].

**Figure 1:**
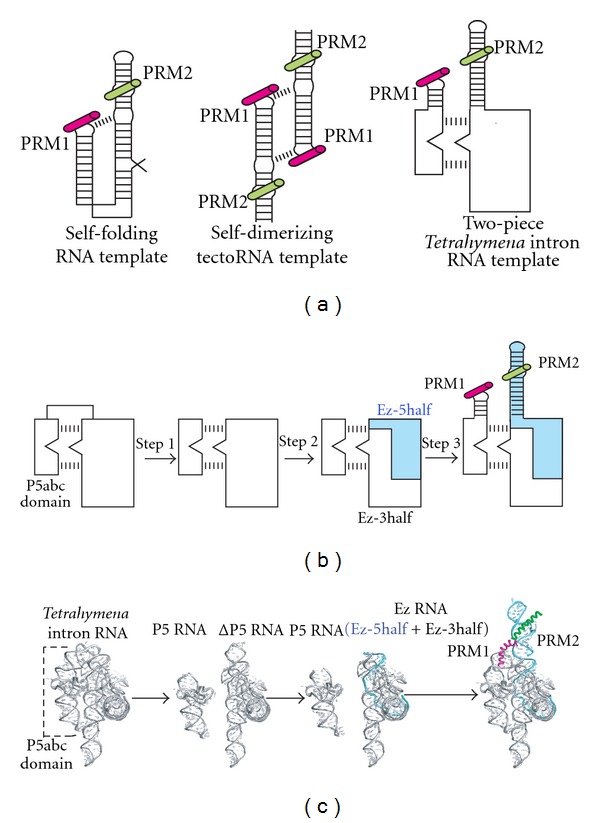
Design of two-piece template RNAs derived from the *Tetrahymena* intron RNA. (a) Self-folding and self-assembling RNA templates for peptide ligation. Dashed lines indicate tertiary interactions that are crucial for 3D structure formation (left) and self-assembly (middle and right) of RNA templates. The substrate peptide-1 and -2 recognized by PRM-1 and -2 are indicated in red (peptide with C-terminal thioester) and green (peptide with N-terminal cysteine), respectively. (b) and (c) 2D (b) and 3D (c) illustrations of three-step construction of two-piece template RNAs from the *Tetrahymena* intron RNA. Step  1: the unimolecular *Tetrahymena* intron (Tet L-30 RNA) was divided into two pieces (P5 RNA and ΔP5 RNA). Step  2: the ΔP5 RNA was divided into two halves (Ez-5half and Ez-3half). Ez-5half is colored pale blue. Step  3: PRM-1 and-2 were added to P5- and Ez-5half, respectively.

These features of multicomponent RNP complexes are not only advantageous in natural molecular evolution but also attractive from the viewpoint of artificial design of functional biomacromolecular systems. Previously, we reported self-folding RNA (Figure 1(a), left) and self-assembling RNA (Figure 1(a), middle) as platforms to design RNA templates for assisting in chemical peptide ligation [15–17]. Previous molecular design for template RNAs had limitations due to the use of the bacteriophage boxB motifs as a dual module for RNA-RNA interaction in the template RNAs and also recognition of the substrate N-peptides (PRM1 in Figure 1(a), left and middle) [16]. This molecular design restricts the scope of substrate peptides.

**Figure 2:**
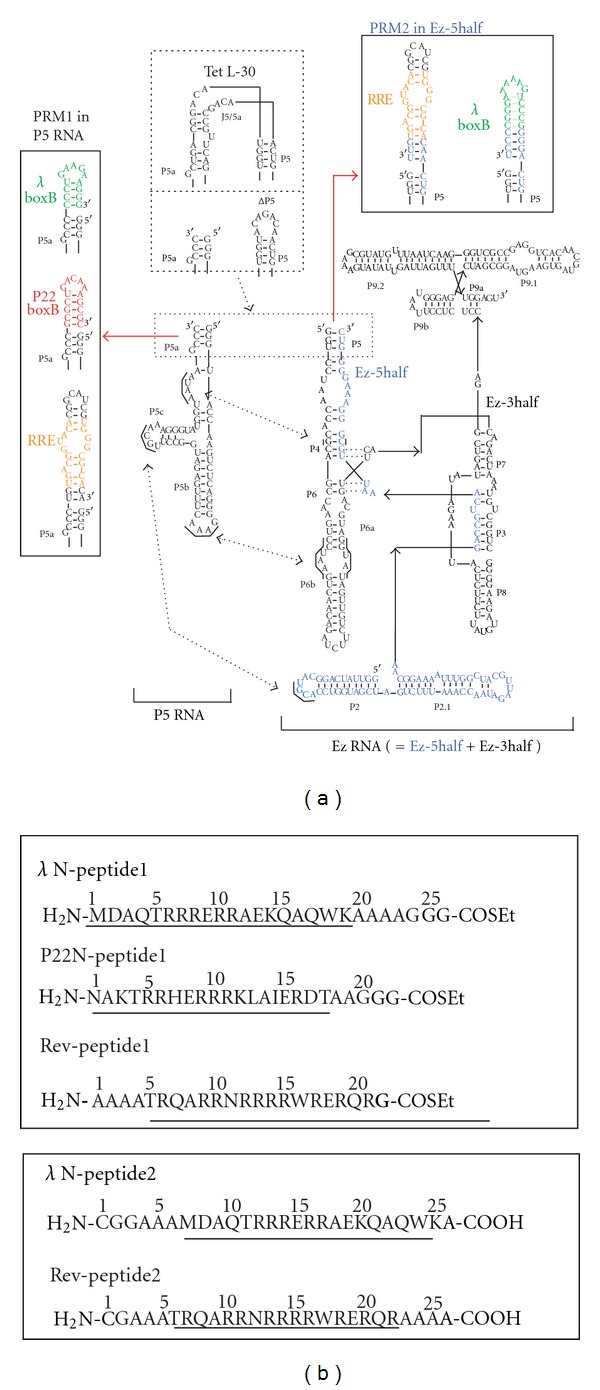
A peptide ligation system with the two-piece *Tetrahymena* RNA template. (a) Secondary structures of the unimolecular *Tetrahymena* intron RNA (Tet L-30) and its two-piece derivatives. Boxes with dotted lines indicate P5/P5a elements at which the parent Tet L-30 was separated to produce a two-piece derivative (P5 : Ez). Dashed arrows indicate the tertiary interactions assembling the P5 and Ez RNA. Boxes with solid lines indicate peptide recognition modules (PRM1 and PRM2) introduced in the P5 RNA and Ez RNA, respectively. (b) Aminoacid sequences of substrate peptides employed for chemical ligation. Underlines indicate the sequences directly responsible for binding with their target RNAs.

In this study, we designed a new template system based on a two-piece derivative of the *Tetrahymena* group I intron RNA (Figure 1(a), right). The *Tetrahymena* group I intron RNA is a unimolecular RNA with modular architecture that exhibits self-splicing activity [18]. Its structural modules can be classified into conserved core modules responsible for catalysis and nonconserved peripheral modules [19]. Peripheral modules, which are not directly involved in the mechanism of catalysis, stabilize the conserved core by organizing a tertiary interaction network that wraps and fixes the core modules (Figures 1(b) and 1(c)) [19–22]. The P5abc domain is a self-folding peripheral element playing crucial roles in stabilization of the core modules [23]. The P5abc domain forms multiple tertiary interactions with the rest of the intron. These interactions are so strong that the function of P5abc is preserved in the two-piece format in which the P5abc domain RNA (P5 RNA) and the rest of the intron (ΔP5 RNA) are physically separated (Figures 1(b), 1(c), and 2(a)). The resulting two RNAs stably form a P5 : ΔP5 complex (Figures 1(b), 1(c), and 2(a)) that is capable of conducting *in vitro* ribozyme functions. We employed this two-piece (P5 : ΔP5) complex as a platform to arrange two peptide recognition modules (Figure 1(a), right) [24, 25]. Although the system designed in this study is still primitive, the RNA design based on the P5 : ΔP5 platform is potentially attractive for development of versatile RNA-based template for chemical peptide ligation.

## 2. Materials and Methods

### 2.1. Molecular Modeling

Molecular models were constructed from the coordinates of the crystal structure of *Tetrahymena* group I intron (Protein Date Bank ID 1X8W) and the NMR structures of the complex of *λ*N-peptide and *λ*boxB RNA (1QFQ), the complex of Rev-peptide and RRE RNA (1ETF), and the complex of P22N-peptide and P22 boxB RNA (1A4T). Molecular modeling was performed by using Discovery Studio (Accelrys Software Inc.) and the PyMOL Molecular Graphics System (Schrödinger, LLC).

### 2.2. Reagents and Instruments

Protected Fmoc aminoacids, *N,N*-diisopropylethylamine (DIEA), HBTU, HOBt, piperidine, HO-CH_2_-PAM resin, and Fmoc-Ala-Alko-PEG resin were purchased from Watanabe Chemical (Hiroshima, Japan). Other reagents and solvents were purchased from Nacalai Tesque (Kyoto, Japan), TCI (Tokyo, Japan), Kishida Chemical (Osaka, Japan), or Kanto Chemical (Tokyo, Japan). Analytical HPLC was performed on a Shimadzu LC-20AB Prominence system. Preparative HPLC was performed on a Senshu Scientific SSC-3465 system. MALDI-TOF-mass spectra were recorded on a Bruker Daltonics Autoflex.

### 2.3. Synthesis of the Peptides Possessing the Carboxy-Terminal Thioesters (Peptides 1)

Synthesis (0.1 mmol scale) was manually carried out using HO-CH_2_-PAM resin (1.0 mmol/g). The first aminoacid (Gly) was introduced as follows. Fmoc-Gly-OH (1.2 mmol) and HOBt (1.2 mmol) were dissolved in dimethylacetamide (DMA) (4 mL). Then *N*, *N*′-dicyclohexylcarbodiimide (DCC) (1.2 mmol) was added to the solution with cooling on the ice for 10 min. To the resulting solution, HO-CH_2_-PAM resin (0.3 mmol) was added and agitated for 17 hours. The resin was then washed with DMF (3 mL) and CH_2_Cl_2_/CH_3_CH_2_OH (1/1) (3 mL) three times, respectively. The resin was further washed with CH_2_Cl_2_ (3 mL) and DMF (3 mL) three times, respectively. The resin was added to benzoic anhydride (1.5 mmol) dissolved in pyridine/DMF (3/1) (3 mL) and agitated for 1 hour. Finally, the resin was washed with DMF (3 mL) and CH_2_Cl_2_ (3 mL) four times. After the introduction of Gly, the second aminoacid was introduced by using HOBt (3 equiv.), HBTU (3 equiv.), and DIEA (6 equiv.). Repetitive removal of the Fmoc groups followed by introduction of protected Fmoc aminoacids was performed according to the standard procedure to give the protected peptide resin. Protected peptide resin (0.033 mmol) in 1.0 mL of dry CH_2_Cl_2_ was stirred for 15 min under argon in a 20 mL flask. In a second flask, 0.67 mL of (CH_3_)_3_Al (2M hexane solution, 0.67 mmol) diluted with 2.33 mL of dry CH_2_Cl_2_ was cooled to 0°C under argon [26, 27]. To this solution, 0.15 mL of ethanethiol (124 mg, 2.0 mmol) was added dropwise, and the resulting mixture was allowed to stir for 15 min at 0°C. This solution was added at once to the suspension of peptide resin in CH_2_Cl_2_. After stirring for 5 hr at room temperature, solvent was removed under reduced pressure. Protected peptide thioester was treated with TFA (2.7 mL) in the presence of* m*-cresol (60 *μ*L), thioanisole (60 *μ*L), ethanedithiol (60 *μ*L), phenol (60 *μ*g), and water (60 *μ*L) for 2 hr at room temperature. The resulting crude peptide was repeatedly purified by HPLC with CosmoSil 5C18-AR-II (20 × 25 mm and 4.6 × 25 mm) under gradient conditions for CH_3_CN in 0.1% aqueous TFA. The fractions corresponding to the desired products were collected and lyophilized. Concentrations of peptide were determined and adjusted by using absorption of amide bonds at 220 nm as follows:

 H_2_N-MDAQTRRRERRAEKQAQWKAAAAGGG-COSCH_2_CH_3_ (*λ*N-peptide1);
 MALDI-TOF-MS: m/z = 2944.282 [M^+^] (calcd. MW = 2944.38);
 H_2_N-AAAATRQARRNRRRRWRERQRG-COSCH_2_CH_3_ (Rev-peptide 1);
 MALDI-TOF-MS: m/z = 2824.679 [M^+^] (calcd. MW = 2825.29);
 H_2_N-NAKTRRHERRRKLAIERDTAAGGG-COSCH_2_CH_3_ (P22N-peptide 1);
 MALDI-TOF-MS: m/z =2766.51 (calcd. MW = 2764.17).

### 2.4. Synthesis of Peptides Possessing Amino-Terminal Cysteins (Peptides-2)

Solid-phase peptide synthesis (0.1 mmol scale) was manually carried out using Fmoc-Ala-Alko-PEG resin (0.25 mmol/g). The resin was treated with 20% piperidine in DMF to remove Fmoc group at the amino-terminus. Then, protected Fmoc aminoacid was introduced to the resin by using with HOBt (3 equiv.), HBTU (3 equiv.), and DIEA (6 equiv.). Repetitive removal of the Fmoc groups followed by introduction of protected Fmoc aminoacids was performed according to the standard procedure to give the protected peptide resin. After the deprotection of Fmoc group of cysteine, the resin was dried under high vacuum. Then, the protected peptide resin was treated with TFA (2.6 mL) in the presence of* m*-cresol (150 *μ*L), thioanisole (150 *μ*L), and ethanedithiol (75 *μ*L) for 1 hr at room temperature. The resulting crude peptide was purified by HPLC with CosmoSil 5C18-AR-II (20 × 25 mm and 4.6 × 25 mm) under gradient conditions for CH_3_CN in 0.1% aqueous TFA. The fractions corresponding to the desired products were collected and lyophilized. Concentrations of peptide were determined and adjusted by using absorption of amide bonds at 220 nm as follows:

 H_2_N-CGGAAAMDAQTRRRERRAEKQAQWKA-COOH (*λ*N-peptide2);
 MALDI-TOF-MS: m/z = 2949.034 [M^+^] (calcd. MW = 2950.38);
 H_2_N-CGAAATRQARRNRRRRWRERQRAAAA-COOH (Rev-peptide2);
 MALDI-TOF-MS: m/z = 3096.007 [M^+^] (calcd. MW = 3095.54).

### 2.5. RNA Preparation

Plasmid encoding the *Tetrahymena* group I intron (pTZ-IVSU) was used as a template for PCR amplification of DNA fragments for transcription of the P5, Ez-5half, Ez-3half, and Tet L-30 RNA [28]. Plasmid encoding the ΔP5abc mutant intron (pL21-ΔP5abc) was used as a template for PCR amplification of a DNA fragment for transcription of the ΔP5 RNA [29]. Peptide recognition modules (PRM-1 and -2) were attached to the template DNAs for the P5- and Ez-5half RNA, respectively by PCR with primers containing the sequence of PRM. Transcription reactions with T7 RNA polymerase were performed according to the published protocol, and transcripts were purified on 6% denaturing polyacrylamide gels. RNAs isolated from the gels were passed through Sephadex G-25 spin columns. The concentrations of RNAs were determined from the intensities of UV absorption at 260 nm.

### 2.6. Peptide Ligation Assay

Chemical ligation of peptides in the presence of RNA molecules was carried out as follows. The P5 RNA, Ez-5half RNA, and Ez-3half RNA were separately denatured in water for 2.5 min at 80°C and then mixed together. To this RNA solution, concentrated reaction buffer (final compositions of the reaction mixture were 40 mM HEPES, pH 8.0, 80 mM KCl, and 10 mM MgCl_2_) was added, and the mixture was warmed for 5 min at 37°C. Reaction was started by adding aqueous solution of substrate peptides (7.5 *μ*M) and 2-mercaptoethanesulfonate (MESNA, final concentration was 1% (w/v)) at 37°C. Samples (20 *μ*L) were quenched with 2 *μ*L of 40% aqueous TFA, and the reaction mixtures were analyzed by HPLC with CosmoSil 5C18-AR-II (4.6 × 25 mm). Gradient conditions for CH_3_CN in 0.1% aqueous TFA were as follows: 5%–32.5% in 22.5 min; flow rate: 1 mL/min. The fractions corresponding to the ligated products were collected and their molecular weights were confirmed by MALDI-TOF-mass spectrometry. All experiments were repeated at least twice. The mean values are shown in the figures in which error bars indicate the minimal and maximal values.

### 2.7. Gel Mobility Retardation Assay

For samples forming complexes, aqueous solutions of the P5 RNA (5 pmol, final concentration 0.5 *μ*M), the 3′ ends of which were labeled with BODIPY fluorophore [30], nonlabeled Ez-5half and Ez-3half RNA (10 pmol, final concentration 1.0 *μ*M) were heated separately at 80°C for 2.5 min. The three RNA solutions where mixed together. To this RNA solution, a 10× concentrated folding buffer was added to adjust the mixture to 50 mM Tris-OAc (pH 7.5) and 10 mM Mg(OAc)_2_ [31]. The resulting mixture was incubated at 37°C for 30 min. After adding 6× concentrated loading buffer consisting of glycerol and XC (0.1%), the samples were loaded onto a 5% nondenaturing polyacrylamide gel (29 : 1 acrylamide : bisacrylamide) containing 50 mM Tris-OAc (pH 7.5) and 10 mM Mg(OAc)_2_. Electrophoresis was carried out at room temperature with 200 V for the initial 5 min and then 75 V for 5 h. The resulting gels were analyzed with a FluoroImager Pharos FX (BioRad, Hercules, CA, USA).

## 3. Results

### 3.1. Molecular Design of the Two-Piece Derivative of the Tetrahymena Intron RNA

To modify the *Tetrahymena* intron RNA as a self-assembling template assisting chemical peptide ligation, a three-step redesign was carried out (Figures 1(b), 1(c), and 2(a)). The first step involves conversion of the parent unimolecular RNA (Tet L-30) to a two-piece derivative consisting of the P5 RNA and the rest of the intron (ΔP5 RNA) as described in the Introduction (Figures 1(b), 1(c), and 2(a)).

The second step involves the introduction of a break in the P5 region of the ΔP5 intron RNA (Figures 1(b), 1(c), and 2(a)). The resulting two fragments (Ez-5half and Ez-3half) reconstitute the ΔP5 RNA through base pairing. The reconstitution of ΔP5 intron RNA (termed Ez in this study) from the two RNA fragments is convenient for installation of the peptide-recognition motif (PRM). In the template DNA for *in vitro* transcription, the introduction of PRM to the Ez-5half fragment of the bimolecular version of the ΔP5 RNA (Ez RNA) can be achieved simply by modifying the PCR primers. On the other hand, for the introduction of a PRM sequence to the corresponding position (the 92nd nucleotide from the 5′ end) of the unimolecular ΔP5 RNA (309 nucleotides), template DNAs need to be prepared using either a reliable but laborious method (plasmid construction using standard recombination techniques) or a convenient but less reliable method (preparation of a double-stranded DNA using multistep PCR to assemble a set of DNA oligonucleotides).

**Figure 3:**
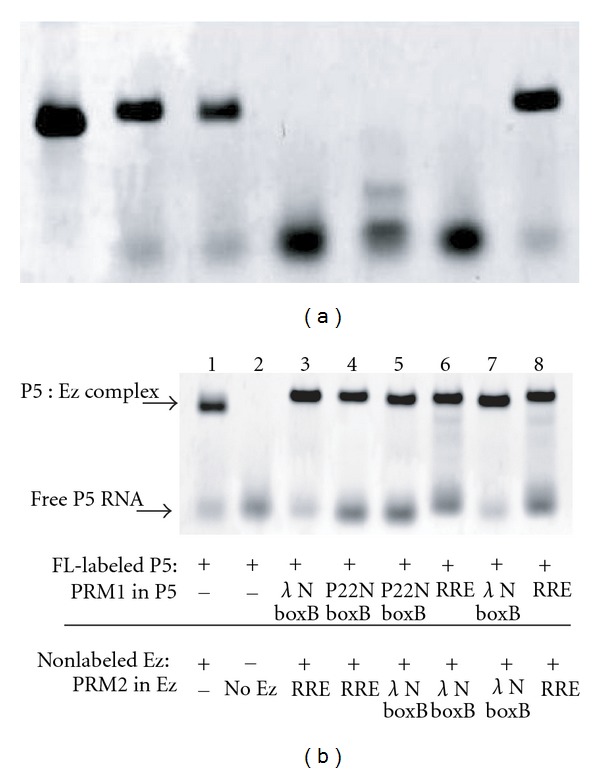
Gel mobility shift assay of the two-piece *Tetrahymena* RNA consisting of three RNA molecules. (a) The Tet L-30 RNA and a bimolecular derivative (P5 + ΔP5) were used as positive controls. Electrophoresis was carried out with 10 mM Mg^2+^ ions. To visualize RNA molecules on the gel, the 3′ ends of FL-labeled RNAs were labeled with BODIPY. (b) Influence of PRM1 and PRM2 on the P5 : Ez complex formation. Electrophoresis was carried out with 10 mM Mg^2+^ ions. To visualize RNA molecules on the gel, the 3′ ends of FL-labeled RNAs were labeled with BODIPY.

Gel mobility shift assay was carried out to confirm reconstitution of the *Tetrahymena* group I intron (Tet L-30) by the three RNAs (P5, Ez-5half, and Ez-3half) (Figure 3(a)) [31]. In the presence of the three RNAs, mobility of the main band was similar to those of the parent unimolecular Tet L-30 RNA and the bimolecular complex (P5 : ΔP5), indicating that the two-piece (P5 and Ez) RNA consisting of three RNA fragments (P5, Ez-5half, and Ez-3half) can be used as a platform RNA (termed the P5 : Ez platform) for chemical peptide ligation.

The third step involves the introduction of two peptide-recognition motifs (PRMs) to the complex (Figures 1(b), 1(c), and 2(a)). The one PRM (PRM1) was introduced to the P5 RNA and the other PRM (PRM2) was introduced to the Ez-5half fragment of the Ez RNA. To determine the versatility of the P5 : Ez platform in chemical peptide ligation, we prepared six template RNA complexes designed for peptide chemical ligations with six combinations of substrate peptides (Figure 2(a)). To confirm that the introduction of PRM1 and PRM2 does not interfere with the complex formation between P5 and Ez, we performed gel mobility shift assay with six template RNA complexes, none of which showed significant reduction of the complex formation (Figure 3(b)). Since the binding between the P5 RNA and the ΔP5 RNA was reported to be highly sensitive to misfolding [32], these results also indicate that neither the folding of P5 and Ez nor their complex formation was disturbed severely by the introduction of PRM1 and PRM2.

### 3.2. Six Combinations of Peptide Chemical Ligations in the Presence of Their Template RNAs

Structure-based design of the template RNAs was carried out by installing two PRMs (PRM1 and PRM2) on the platform P5 : Ez complex (Figure 1). Each PRM1 captures its target substrate (peptide1) bearing the reactive thioester group in its C-terminus. Each PRM2 captures its target substrate (peptide2) bearing cysteine in its N-terminus.

To employ three RNA-binding peptides (*λ*N-peptide1, P22N-peptide1, and Rev-peptide1, Figure 2(b)) as the substrate peptide-1, we designed three P5 RNA derivatives with PRM1s for substrate peptides1 (*λ*boxB, P22boxB, and RRE). As the substrate peptides2 recognized by the Ez RNA, we used two peptides (*λ*N-peptide2 and Rev-peptide2, Figure 2(b)). Their target *λ*boxB and RRE motifs were installed to the Ez RNA as PRM2s (Figure 2(a)).

**Figure 4:**
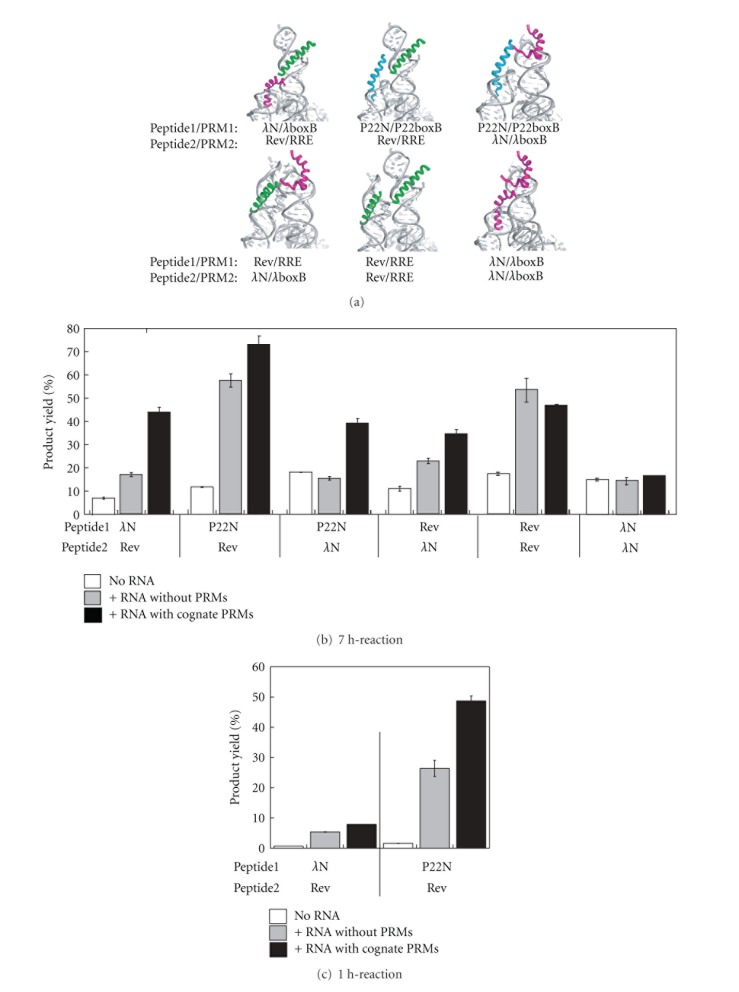
Chemical ligation of six sets the substrate pairings in the presence of their cognate RNA templates. (a) 3D models of peptide recognition modules in substrate-template RNP complexes. RNA elements including PRM-1 and -2 are indicated in gray. Substrate peptides were indicated in red (*λ*N-peptide), green (rev-peptide), and blue (P22N-peptide), respectively. (b and c) Product yields of 7-h chemical ligation (with 7.5 *μ*M each peptide) in the presence of 10 mM Mg^2+^ ions. Reactions of the substrate peptides in the absence of template RNA are shown by white bars. Reactions in the presence of RNA templates having PRMs and lacking PRMs are shown by black and gray bars, respectively. (c) Product yields of 1-h chemical ligation (with 7.5 *μ*M each peptide) in the presence of 10 mM Mg^2+^ ions. Reactions without RNA are shown by white bars. Reactions with RNA templates having PRMs and lacking PRMs are shown by black and gray bars, respectively.

Basal activities of six combinations of the substrate peptide-1 and -2 were investigated (Figure 4). In the absence of template RNAs, products yields in 7 h reaction of chemical ligations with six combinations of substrates (7.5 *μ*M each) were around 10–20% (Figure 4(b)).

To evaluate the ability of template RNA derived from the platform complex (P5 : Ez), we designed and examined six combinations of peptide ligation reactions based on the model 3D structures of the substrate-template complexes (Figure 4(a)). In the presence of stoichiometric amounts of the cognate template RNA complex (7.5 *μ*M), product yields of the four reactions were improved to around 40% (Figure 4(b)). The reaction between P22N-peptide1 and Rev-peptide2 exhibited remarkable improvement of the product yield that reached 73%. On the other hand, the reaction between *λ*N-peptide1 and *λ*N-peptide2 was not promoted by the template RNA (Figure 4(b)).

To determine whether the improvement of the reaction is due to the complex formation modeled in Figure 4(a), control reactions were carried out using the parent P5 and Ez RNAs lacking PRM1 and PRM2. In the reactions between Rev-peptide1 and Rev-peptide2 and between *λ*N-peptide1 and *λ*N-peptide2 (Figure 4(b)), PRMs gave no positive effect (Figure 4(b)). These results are predictable because these templates capture the two substrates in a productive manner (peptide1/PRM1 + peptide2/PRM2) and inhibitive manners (peptide2/PRM1 + peptide1/PRM2, peptide1/PRM1 + peptide1/PRM2, and peptide2/PRM1 + peptide2/PRM2). Since the yield of the reaction between P22N-peptide1 and Rev-peptide2 (58%) was close to the yield in the presence of the cognate template RNA (73%), the template effect for this reaction was compared in the early phase of the reaction (1-h reaction, Figure 4(c)). The product yield of the cognate template RNA was twofold higher than the yield with the control RNA. On the other hand, the effect of the control RNA on the reaction between P22N-peptide1 and Rev-peptide2 was more efficient than the effect of the cognate template RNA on the reaction of *λ*N-peptide1 and Rev-peptide2 (Figure 4(c)).

### 3.3. Mutational Analysis of the Contribution of the Two-Piece Template RNA on the Ligation between *λ*N-Peptide1 and Rev-Peptide2

Among the six combinations of chemical peptide ligations, the reaction between *λ*N-peptide1 and Rev-peptide2 was chosen for further investigation of the template ability of the self-assembling RNA derived from the P5 : Ez complex.

**Figure 5:**
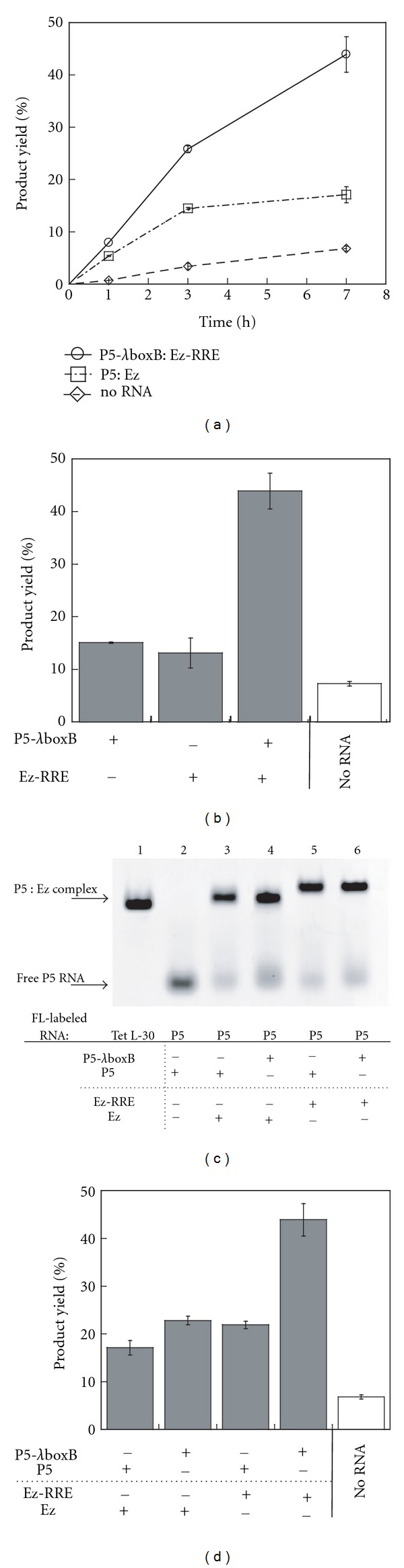
Dissection of the template RNA complex (P5-*λ*boxB : Ez-RRE) for the chemical ligation between *λ*N-peptide1 and rev-peptide2. (a) Time course of ligation reaction (with 7.5 *μ*M each peptide) in the presence of 10 mM Mg^2+^. Reactions were carried out with template RNA having PRM (P5-*λ*boxB : Ez-RRE) and lacking PRM (P5 : Ez), and also without RNA template. (b) The contribution of the template subunit having PRM (P5-*λ*boxB and Ez-RRE subunits). Reactions were carried out for 7 h in the presence of 10 mM Mg^2+^ ions. (c) Effects of PRMs on the P5 : Ez complex formation. Mobility shift assay was carried out with native gal electrophoresis in the presence of 10 mM Mg^2+^ ions. To visualize RNA molecules on the gel, the 3′ ends of FL-labeled RNAs were labeled with BODIPY. (d) The contribution of PRMs in the template ability of the two-piece RNA complex (P5-*λ*boxB : Ez-RRE). Reactions were carried out for 7 h in the presence of 10 mM Mg^2+^ ions.

Reaction time courses indicated that the ligated product was produced through (i) intrinsic reactivity of the two peptides, (ii) assistance of RNA in a PRM-independent manner, or (iii) assistance of RNA in a PRM-dependent manner (Figure 5(a)). To evaluate the contributions of (ii) and (iii), we systematically prepared mutant complexes and investigated their effects. To determine the importance of two PRMs being a pair in the PRM-dependent promotion of the ligation, we examined the ligation reaction in the presence of only the P5-*λ*boxB RNA or the Ez-RRE RNA (Figure 5(b)). The product yields of the two reactions (15% and 13%) were lower than the yield with cognate complex (44%) and close to the yield with the platform P5 : Ez complex (17%). To further investigate the effect of PRM, we removed one PRM from the template complex. Complex formation between P5 and Ez was not affected by the number of PRM in the P5 : Ez complex (Figure 5(c)). The resulting two complexes lacking either PRM1 or PRM2 served as templates much less effectively than the complex with two PRMs, and their effects were comparable to that of the platform complex (P5 : Ez) (Figure 5(d)). These results indicated that the PRM-dependent enhancement was achieved by cooperative effects of PRM1 (*λ*boxB motif) and PRM2 (RRE motif). The product yields in the presence of the mutant complexes possessing one PRM (P5-*λ*boxB : Ez and P5 : Ez-RRE in Figure 5(d)) were slightly higher than those in the presence of P5-*λ*boxB or Ez-RRE alone (Figure 5(b)). This difference may reflect the PRM-independent enhancement contributed by the Ez and P5 components in the mutant P5-*λ*boxB : Ez and P5 : Ez-RRE complexes, respectively.

## 4. Discussion

In this study, we employed the two-piece derivative of the *Tetrahymena* intron RNA (P5 : ΔP5 complex) as a self-assembling platform for a template RNA to assist peptide ligation. The structural organization of the P5 : ΔP5 platform ensures structural independence between the modules for RNA-RNA assembly and the modules for recognition of substrate peptides (PRM), suggesting the versatility and flexibility of the P5 : ΔP5 platform in molecular design. This study confirmed the versatility of the P5 : ΔP5 platform for template design because six types of template RNA complex were designed and their template capabilities were tested experimentally. On the other hand, this study also revealed the current limitation of the P5 : ΔP5 platform. In the case of the reaction between *λ*N-peptide1 and Rev-peptide2, the PRM-dependent facilitation by the P5 : Ez complexes possessing two PRMs was similar to the facilitation by the self-folding P4–P6 RNA with two PRMs [16]. The PRM-independent facilitation by the parent P5 : Ez platform, however, was more efficient than those by the previous platforms based on the self-folding RNAs [16]. The negatively charged surface of 3D-RNAs electrostatically accumulates positively charged substrate peptides [33, 34]. This effect may be stronger in the P5 : ΔP5 platform than in previous platforms because of the considerably larger surface area of the P5 : ΔP5 platform. This property is undesirable if the P5 : ΔP5-based RNA is applied as a template to conduct PRM-dependent ligation of the cognate substrate pair in the presence of non-cognate substrates. Therefore, reduction of the surface size of 3D-RNA may be a strategy to suppress PRM-independent facilitation of peptide ligation. In the P5 : ΔP5 platform, however, most of the core and peripheral modules contribute to self-assembly of P5 and ΔP5 and also maintenance of its 3D structure. Addition of cosolute molecule to the buffer solution may be an alternative approach [35, 36]. Positively charged small molecules or polymers may competitively block the substrate peptide to assemble with the platform region of the template RNA, thus competitively suppressing the PRM-independent reaction promotion. On the other hand, an appropriate amount of cationic cosolute may not inhibit specific binding between peptides and their target PRMs, resulting in preservation of PRM-dependent reaction.

An unexpected finding of the present study was the exceptionally efficient RNA assistance of ligations between P22N-peptide1 and Rev-peptide2 and between Rev-peptide1 and Rev-peptide2 without PRMs (Figure 4(b)). In contrast to the two reactions, RNA templates gave no positive effect on the reaction between *λ*N-peptide1 and *λ*N-peptide2 (Figure 4(b)). No PRM-independent facilitation was observed in the reaction between P22N-peptide1 and *λ*N-peptide2 (Figure 4(b)). These results suggest that the PRM-independent reaction promotion by the P5 : ΔP5 platform depends on the combination of RNA-binding peptides. Rev-peptide and P22N-peptide seem more congenial to the RNA-promoted chemical ligation than *λ*N-peptide (Figure 4). Although the molecular basis underlying these results remains to be elucidated, it is important to see whether apparently different results originate from the physical property of each peptide. *λ*N-peptide is less hydrophilic than P22N- and Rev-peptides (Figure 2(b)), and P22N-peptide has markedly higher affinity to cognate and noncognate RNA motifs than *λ*N- and Rev-peptides [37]. Thus, it is also important to determine whether the possible approaches to suppress PRM-independent reaction promotion are effective on the reaction between P22N-peptide1 and Rev-peptide2.
